# PREVALENCE AND PREDICTORS OF CHANGES IN BOWEL HABITS AFTER LAPAROSCOPIC CHOLECYSTECTOMY

**DOI:** 10.1590/0102-6720201700010002

**Published:** 2017

**Authors:** Leonardo de Mello DEL GRANDE, Luis Fernando Paes LEME, Francisco Pimenta MARQUES, Andressa Teruya RAMOS, Paula Teruya RAMOS, Felipe Araújo de SOUZA

**Affiliations:** Edmundo Vasconcelos Hospital Complex, São Paulo, SP, Brazil

**Keywords:** Cholecystectomy, Bowel habits, Post-cholecystectomy syndrome, Diarrhea.

## Abstract

**Background:**

The incidence of cholecystolithiasis is approximately 15% of the population. It is believed that between 30-40% of cholecystectomy patients have symptoms after surgery, being changes in bowel habits the most common among them.

**Aim:**

1) Defining the prevalence, and 2) identifying predictors of changes in bowel habits after laparoscopic cholecystectomy.

**Methods:**

This is a retrospective cross-sectional study with an initial sample of 150 patients diagnosed with cholecystolithiasis operated between July and September 2014. Patients were submitted to a questionnaire about the presence of gastrointestinal symptoms and changes in stools consistency before and after the surgical procedure. They were divided into two groups (with or without changes in bowel habits) being combined with the following variables: high blood pressure, body mass index, hypothyroidism, adherence to postoperative dietary orientations, previous abdominal and bariatric surgery.

**Results:**

The prevalence of changes in bowel habits in the study population was 35.1%. The association between it and gastrointestinal symptoms was demonstrated to be statistically significant (*‰*2=7.981; p=0.005), and people who did not have gastrointestinal symptoms had 2.34 times the odds of not presenting changes in bowel habits. None of the other investigated factors had shown to be a predictor of risk for post-cholecystectomy changes in bowel habits.

**Conclusion:**

1) There was a high prevalence of changes in bowel habits, and 2) there was association between changes in bowel habits and the presence of gastrointestinal symptoms.

## RESUMO

**Racional:**

A incidência da colecistopatia calculosa é de aproximadamente 15% da população brasileira. Acredita-se que entre 30-40% dos pacientes colecistectomizados apresentem sintomas pós-operatório, sendo a alteração do hábito intestinal o mais comum.

**Objetivo:**

1) Determinar a prevalência, e 2) identificar preditores de mudanças do hábito intestinal pós-colecistectomia videolaparoscópica.

**Métodos:**

Estudo transversal retrospectivo com amostra inicial de 150 pacientes diagnosticados com colecistopatia calculosa, operados entre julho e setembro de 2014. Os pacientes foram submetidos a um questionário sobre a presença de sintomas gastrointestinais após a operação, e a alterações do aspecto das fezes antes e depois do procedimento. A amostra foi dividida em dois grupos (com ou sem mudanças do hábito intestinal) sendo realizadas associações com as seguintes variáveis: hipertensão arterial, IMC, hipotireoidismo, seguimento de dieta no pós-operatório, operação abdominal prévia e operação bariátrica.

**Resultados:**

A prevalência de mudanças do hábito intestinal na população estudada foi de 35,1%. A associação entre elas e sintomas gastrointestinais demonstrou-se estatisticamente significativa (*‰*2=7,981; p=0,005), sendo que as pessoas que não apresentavam os sintomas tinham 2,34 vezes mais chances de não apresentarem mudanças do hábito intestinal. Nenhum dos demais fatores investigados demonstrou ser preditor de risco para mudanças do hábito intestinal pós-colecistectomia.

**Conclusões:**

1) Observou-se alta prevalência de mudanças do hábito intestinal, e 2) houve associação entre mudança do hábito intestinal e a presença de sintomas gastrointestinais.

## INTRODUCTION

It is estimated that gastrointestinal diseases affect circa 60 million Americans every year. In 2004, it was estimated that 4.6 million hospital admissions, 72 million outpatient visits, and 236 million deaths were related to gastrointestinal diseases^14^.

Cholecystolithiasis affects approximately 20% of the Western adult population, of which 15% become symptomatic^9^. In Brazil, in 2014, 130,000 cholecystectomy procedures were performed by the Brazilian Public Healthcare System (Sistema Único de Saúde), 21 million of which in the State of São Paulo^11^.

Since its introduction by Mühe^12^ in 1986, laparoscopic cholecystectomy became widely popular and is currently considered the treatment of choice for cholecystolithiasis. This alternative has several advantages when compared to laparotomy. All of them have already been well documented in the literature, such as shorter hospital stays, less post-surgery pain, and decreased morbidity, besides having the best cost-benefit ratio^7^. The dissemination of laparoscopy-oriented diagnosis and surgical procedures enabled an expressive increase in surgical approaches to cholecystectomy, especially if they have no complications related to lithiasis^16^.

Post-cholecystectomy gastrointestinal symptoms are usually unspecific and mild, such as flatulence, nausea, eructation, indigestion, and changes in bowel habits (CBH)^2^ ^,^ ^10^ ^,^ ^13^. The most important change in bowel habits after cholecystectomy is diarrhea, the prevalence of which is reported to be between 0.9 to 35.6% and one of the most distressing post-surgery sequelae^4^ ^,^ ^5^. Besides the significant number of symptomatic patients, there are no reports in the literature of how to determine which patients are going to develop CBH.

Minor digestive disorders in the weeks after surgery are of variable intensity and do not lead to, except for extreme cases, hospital admissions. Diarrhea or soft stools are frequently reported and are due to the adaptation period of the biliary tree to the lack of a reservoir (the gall bladder), gradually improving after a period. Only a limited number of patients complain about constipation, which is probably related to their inactivity and dietary restrictions recommended by some surgeons^20^.

Physiopathology of CBH is a controversial subject. The literature usually attribute it to alterations in the enterohepatic circulation of bile acids. Cholecystectomy removes the major reservoir of bile acids, what causes an increase in the time it is kept in contact with bowel mucus between meals^4^. Bile acids are subject to greater bacterial dihydroxylation, hampering their intestinal absorption, causing bigger quantities of which to enter the large intestine, which is believed to be the leading cause of post-surgery CBH^8^ ^,^ ^15^.

Due to the increase in the number of laparoscopic cholecystectomy, information on its prevalence, mechanisms and predisposing factors for post- cholecystectomy CBH is increasingly important to manage patients' expectations, to obtain their consent and to enable postoperative care^5^.

This study aims at: 1) defining the prevalence, and 2) identifying predictors of changes in bowel habits after laparoscopic cholecystectomy.

## METHODS

This is a retrospective cross-sectional study with an initial sample of 150 patients diagnosed with cholecystolithiasis operated between July and September 2014 at Complexo Hospitalar Edmundo Vasconcelos, in São Paulo, SP, Brazil. This study received the ethics approval of the Research Ethics Committee of this institution, and verbal consent was obtained from research participants before telephone interviews.

All patients that accepted to be part of the study were included. Those that do not answered after four call attempts in different days and alternate periods of the day, those that refused to join, and a patient with gall bladder agenesis were excluded.

A previously validated questionnaire^4^ was modified to fit the purpose of this study and used to identify the effects of laparoscopic cholecystectomy on CBH. The questionnaire was comprised by detailed questions about bowel habits before and after the surgical procedure, postoperative dietary orientations, comorbidities, previous abdominal surgery, and randomly reported symptoms. Body Mass Index (BMI) in the day of surgery was calculated using the information available in the Institution's information system.

**FIGURE 1:**
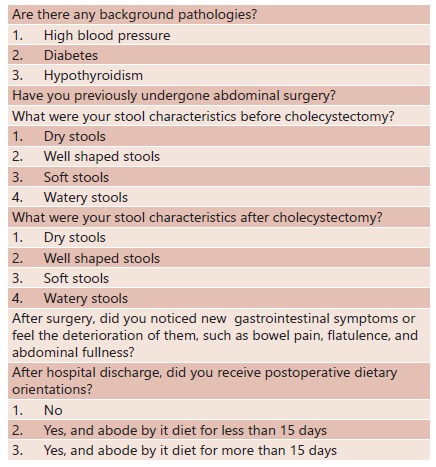
Questionnaire used in this research

A stools consistency scale was applied according to the following: 1 dry stools; 2 well shaped stools; 3 soft stools; and 4 watery stools. Participants were divided in two groups, those that had CBH (deterioration of stools consistency) and those that did not have it, which means they have kept the same stool consistency before and after laparoscopic cholecystectomy. Adherence to postoperative dietary orientations was determined if participants abided by anti-fermentation and low-fat diets for 15 days or more.

After the division of research participants in these two groups, was calculated their qui-square aiming at investigating the association between the following variables: 1) CBH vs. diabetes (presence or absence); 2) CBH vs. gastrointestinal symptoms (GS, presence or absence); 3) CBH vs. systemic high blood pressure (HBP, presence or absence); 4) CBH vs. BMI (below or above the overweight threshold); 5) CBH vs. hypothyroidism (presence or absence); 6) CBH vs. adherence to postoperative dietary orientations (less than 15 days, more than 15 days, or no dietary orientations); 7) CBH vs. abdominal surgery (previous or no bariatric surgery); and, 8) CBH vs. gender (male or female).

### Statistical analysis

Qui-square tests were performed separately for each association. P values below 0.05 (p*<*0.05) were considered statistically significant. For those associations considered statistically significant, was calculated their odds-ratio (OR). Odds ratio relates to the estimate of effect of the association found statistically relevant. The OR was calculated based on the probability that an event occurs divided by the probability that it would not occur. Student t-tests were performed aiming at assessing the differences of age between participants that had CBH and gastrointestinal symptoms. T-tests used resampling procedures (bootstrapping; 1,000 resampling; confidence interval of 99%). Resampling procedures were used in order to ensure the highest level of reliability of these results, besides correcting possible distribution deviances in the normality of data and incompatibility in groups' sizes, apart from introducing a confidence interval of 99% for differences in means^6^.

## RESULTS

There were 111 research participants, 70% of which were women, from 18-84 years of age (46.11±14.61). With respect to women, age varied between 18-79 years of age (43.73±14.02), and to men, it varied between 27-84 years of age (51,84±14,61).

Among the group of participants, for 64.9% (n=72) there was no changes in stools consistency before and after the surgery, while there was a deterioration of stools consistency for 35.1% (n=39). There were no changes in stool consistency for 60.6% (n=20) of men, and there were deteriorations in stools consistency for 39.4% (n=13) of them. For women, 66.7% (n=52) had no changes in stool consistency, while 33.3% (n=26) of them had deteriorations in stools consistency.

Table 1 relates the analyzed variables with their respective qui-square results and p-values.

**TABLE 1:** Analyzed variables

Associations	Qui-Square	p
CBH X Diabetes	0.534	0.465
CBH X Gastrointestinal Symptoms	7.981	0.005
CBH X HBP	0.002	0.968
CBH X BMI	0.214	0.644
CBH X Hypothyroidism	0.012	0.912
CBH X Adherence to Dietary Orientations	0.421	0.810
CBH X Abdominal Surgery	0.911	0.634
CBH X Gender	0.374	0.541

The only statistically significant analyzed variable was the association between CBH and gastrointestinal symptoms (GS). The OR demonstrated that people who did not had GS had 2.34 more chances to show no changes in stools consistency before and after surgery, when compared to those that had GS.

Student t-test was performed to evaluate if there were significant differences in the age of participants who have and have not reported CBH and GS. Table 2 shows the descriptive and inferential results of this test. It demonstrates that, for both variables, there were no statistically significant differences with reference to the age of participants.

**TABLE 2:** Differences in the age of participants

Groups		Descriptive Data			Inferential Data		
		Means	Standard deviation	Confidence interval for Means (99%)			Student t-Test	
				Lower limit	Upper limit	t	p
CBH	Consistência igual	46,944	14,469	42,4478	51,4097	0,782	0,436
	Consistência diminuída	44,666	14,963	38,4161	50,8849		
GS	Sim	44,950	13,299	39,5001	50,3563	0,710	0,479
	Não	47,014	15,380	42,9573	51,9388		

## DISCUSSION

This study has demonstrated that changes in bowel habits, analyzed by the deterioration in stools consistency, affects approximately 35% of laparoscopic cholecystectomy patients, as described by current literature^4^ ^,^ ^5^. Due to the difficulty in defining post-cholecystectomy diarrhea using simple concepts, was chosen to study it according to the deterioration of stools consistency and, as such, changes in bowel habits.

The use of postoperative low-fat diet after cholecystectomy is controversial in the literature and among surgeons. Despite there are no documented improvements in the symptoms due to adhering to a low-fat diet, many physicians prescribe it and, when they don't, many patients keep this diet after surgery for fearing consuming fat-rich food, once they are related to intolerance and preoperative symptoms^10^. Previous studies demonstrated benefits of a low-fat diet in reducing the incidence of diarrhea during the first week after surgery^5^. This study does not demonstrate a statistically significant association between adherence to a low-fat postoperative diet and changes in bowel habits, which reaffirms findings of certain studies that assess the benefits of recommending a low-fat postoperative diet^10^.

The prevalence of diarrhea is higher in obese patients when compared to the population that has an adequate weight. A population survey in Rochester, MN, that has 2,660 participants demonstrated that the prevalence of diarrhea in obese individuals reaches 30% of participants, comparing to a prevalence of 17% in the control group [OR=2.7 (95% CI 1.1-6.8)]. The higher prevalence of diarrhea can be associated with changes in bile acids, resulting in bile acid diarrhea^3^. Similar studies were replicated in Australia and New Zealand, which proposed that the alteration could be due to the colonic transit and/or an increase in intestinal mucus permeability^17^ ^,^ ^18^. Obesity could also be associated with an increase in fecal calprotectin, a marker for bowel inflammation. Drugs used by obese individuals, such as metformin for diabetes mellitus type 2 or polycystic ovary, could also cause diarrhea^1^.

This study did not show any association between changes in bowel habits and obesity or diabetes. Hypothyroidism and high blood pressure also did not demonstrate any association with diarrhea and other gastrointestinal symptoms, as demonstrated in the literature.

The association found between CBH and GS is evidence that there are other factors associated with lasting postoperative symptoms of laparoscopic cholecystectomy, and not only the absence of the gall bladder. The literature shows reports of studies that present the hypothesis of mood alterations influencing gastrointestinal symptoms^19^.

There is no objective method for evaluating diarrhea and other changes in bowel habits. Hence, applying a questionnaire is a subjective method that could under- or overestimate symptoms, making it harder to assess and interpret research findings.

New prospective studies are indispensable to define the risk factors for CBH after laparoscopic cholecystectomy. While they are not identified, surgeons and patients need to be aware that such alterations are frequent and that there is no means to foresee it.

## CONCLUSION

1) There is a high prevalence of changes in bowel habits in the study's sample, being present in 35.1% of the patients participating in the research; 2) there is an association between changes in bowel habits and the presence of gastrointestinal symptoms.
